# Intravitreal Medications for Retinal Vein Occlusion: Systematic Review and Meta-analysis

**DOI:** 10.18502/jovr.v14i3.4791

**Published:** 2019-07-18

**Authors:** Alireza Lashay, Hamid Riazi-Esfahani, Masoud Mirghorbani, Mehdi Yaseri

**Affiliations:** ^1^ Translational Ophthalmology Research Center, Farabi Eye Hospital, Tehran University of Medical Sciences, Tehran, Iran; ^2^ Department of Epidemiology and Biostatistics, Tehran University of Medical Sciences, Tehran, Iran

**Keywords:** Anti-vascular Endothelial Growth Factor, Dexamethasone, Retinal Vein Occlusion, Triamcinolone

## Abstract

**Purpose:**

To evaluate the outcomes of different intravitreal injections for the treatment of retinal vein occlusion including central retinal vein occlusion (CRVO) and branch retinal vein occlusion (BRVO).

**Methods:**

PubMed, Cochrane, the metaRegister of ControlledTrials, and ClinicalTrials were searched for intravitreal anti-Vascular Endothelial Growth Factor (VEGF) and steroids with relevant keywords and date limitation of 2009-2018. Meta-analysis was performed on studies that met the defined inclusion criteria. Main outcomes were visual acuity (VA) and central macular thickness (CMT).

**Results:**

Out of 681 studies, 36 articles (including 21 reporting CRVO and 15 dealing with BRVO) were selected for systematic review. All five intravitreal drugs including triamcinolone, dexamethasone, ranibizumab, bevacizumab, and aflibercept showed improvement of CMT and VA as compared to placebo or laser treatment. Six randomized controlled trials were selected for meta-analysis in RVO patients. The pooled mean difference of visual improvement between sham and ranibizumab was 12.7 Early Treatment for Diabetic Retinopathy Study (ETDRS) letters (95%CI: 11.00 to 13.2), and the pooled mean difference in CMT reduction was 221μm (95%CI: 153 to 284); both changes were significantly in favor of ranibizumab. The pooled mean difference of visual improvement between bevacizumab and triamcinolone was 5.3 ETDRS letters in favor of bevacizumab (95%CI: 16 μm to 17.5 μm). Triamcinolone led to 68.1 μm greater CMT reduction than bevacizumab (95%CI: 58 μm to 76 μm). However, none of these differences were statistically significant.

**Conclusion:**

Treatment with anti-VEGF agents in RVO is superior to observation. No significant difference was seen between the eyes treated with bevacizumab or triamcinolone based on these results.

##  INTRODUCTION

Retinal vein occlusion (RVO) is caused by thromosis in central, hemi-central, or branch retinal veins.^[1]^ In central retinal vein occlusion (CRVO), the obstruction usually occurs at the level of the lamina cribrosa, while in branch retinal vein occlusion (BRVO), it involves a branch of the central retinal vein.^[1,2,3]^ The clinical manifestations of RVO is largely related to the secondary elevation of Vascular Endothelial Growth Factor (VEGF) levels in the vitreous and retina due to retinal ischemia.^[1]^ The following conditions have been variably reported as associations of RVOs: systemic hypertension, diabetes mellitus, hyperlipidemia, hyper-homocysteinemia, blood coagulation disorders, systemic inflammatory disorders, glaucoma, short axial length, and high body mass index.^[2,3,4,5,6]^ Two major consequences of RVO which lead to decreased visual acuity (VA) are macular edema (ME) and retinal ischemia. In eyes with non-ischemic CRVO, VA improves significantly following resolution of ME.^[4,6,7]^ However, in the eyes with ischemic CRVO, no significant association has been found between the presence or absence of ME and improvement in VA due to permanent damage to macular ganglion cells.^[7]^ Other factors may also affect the natural history of CRVO. Demographic factors such as age or male gender, systemic factors including vascular risk factors or high levels of blood hematocrit, and ocular factors such as macular pigmentary change, epiretinal-membrane formation following long-standing ME, retinociliary collaterals, and glaucoma have been reported to be associated with poor functional outcomes.^[8]^ The development of anterior segment neovascularization also has a detrimental effect on visual outcomes.^[7,8]^ Based on the Branch Retinal Vein Occlusion Study (BVOS) study, the visual prognosis in BRVO is better than CRVO.^[9]^ Therefore, it is not surprising to observe relatively good outcomes in the control group of large randomized controlled trials (RCTs) such as the Study on the Efficacy and Safety of Ranibizumab Injection in Patients With Macular Edema Secondary to BRVO (BRAVO). Visual acuity improvement in eyes with macular BRVO is usually more marked than eyes with major BRVO.^[10]^ Ischemic insult to macular ganglion cells, pigmentary degeneration, and development of epiretinal membrane may adversely affect the visual outcome in BRVO.^[11]^ Various treatments have been proposed for the management of RVO-related ME and many RCTs have been designed to compare these therapies and their long-term outcomes. The selection of the most efficacious therapy providing the best outcome in clinical practice necessitates ophthalmologists to be updated on the results of recent trials and adopt a comprehensive approach toward the patient. In the current study, we aim to provide an update on recent trials addressing the management of RVO-related ME, compare the outcomes of these trials, and perform a meta-analysis on studies with similar arms according to the pre-defined inclusion and exclusion criteria.

##  METHODS

This systematic review and meta-analysis complies with the Preferred Reporting Items for Systematic Reviews and Meta-analyses (PRISMA) - 2009 rules.

###  Search Methods for Identifying the Studies

Two investigators (HR and MM) participated in the literature search via PubMed, Cochrane, the metaRegister of ControlledTrials, and ClinicalTrials in English language with date limitation of 2009-2018. In the search strategy which was last conducted on March 21, 2018, we used the MeSH term “retinal vein occlusion” and the words “*RVO”, “*vitreal”, “*VEGF*”, “bevacizumab”, “Avastin”, “ranibizumab”, “Lucentis”, “aflibercept”, “Eylea”, “triamcinolone”, “implant”, and “Ozurdex”. It should be noted that we only searched for the most popular agents in clinical practice and not the miscellaneous drugs presented in the case reports or case series.

###  Selection of Studies for Systematic Review

All titles and abstracts were independently screened by two co-authors (HR and MM) and the potential relevance was judged. Any disagreement between the two authors was referred to the corresponding author (AL) for final decision. The final list of included studies was re-evaluated to ensure proper study selection. Only papers with full-texts or abstracts in English were selected.

###  Data Collection

Data was collected separately by HR and MM from all included studies:

• First and corresponding authors, journal, year, main criteria for inclusion in the study, any exclusion criteria, number of eyes, treatment arms, and length of the study.
• Means and standard deviations of changes in corrected VA and central macular thickness (CMT) changes from baseline.

Each article was evaluated carefully and rated by the panel (HR, MM, and MY) according to the level of evidence provided by the study. The level of evidence was assigned to each study according to the latest guidelines of the British Centre for Evidence-Based Medicine;^[12]^

• Level I: well-conducted and designed randomized clinical trials
• Level II: lower-quality randomized, well-designed case-control, and cohort studies
• Level III: lower-quality cohort and case-control studies and case series

###  Outcome Measurement

The mean change of VA from baseline was the primary outcome measure. The secondary outcomes included: (1) The proportion of patients gaining 15 Early Treatment for Diabetic Retinopathy Study (ETDRS) letters or more compared to baseline at different time points; (2) Mean change of CMT on optical coherence tomography (OCT) from baseline as the anatomical outcome measure.

###  Meta-analysis: Inclusion and Exclusion Criteria

Studies were included in the meta-analysis if they met the following criteria: (1) randomized controlled clinical trials with level I of evidence, (2) mean follow-up of six months or more, (3) comparing anti-VEGF or intravitreal corticosteroid with placebo or laser treatment for ME due to CRVO or BRVO, (4) providing the proportion that gained 15 ETDRS letters or more, (5) providing changes of VA and CMT in the treatment and sham groups for calculating mean difference (MD), odds ratio (OR), and 95% confidence interval (95% CI). Meta-analysis was performed on studies with level I evidence with comparable arms and methods. Studies were excluded from meta-analysis if they were retrospective, non-controlled, non-randomized, or were not in English. We chose six months as the minimum time point for meta-analysis. The mean change in VA and CMT was measured as a continuous variable and calculated as MD with 95% CI.

###  Methodological Quality/Risk of Bias Assessment

The methodological efficiency of studies was evaluated for the quality based on the modified Jadad scoring system.^[13,14]^ Through this assessment tool, we evaluated three main study characteristics including randomization, blinding, and participant dropout. Studies with Jadad score of three points or more were considered as high-quality studies. Also, a risk of bias summary was provided by each data collector separately including selection bias, detection bias, and attrition bias in order to assess various potential sources of systemic-bias.

###  Heterogeneity Assessment

Both clinical and methodological heterogeneities were assessed for meta-analysis. We considered I2 values more than 60% to indicate substantial statistical heterogeneity. The Random-effects model was used for meta-analysis.

###  Statistical Analysis

All analyses were performed using the Stata (StataCorp. 2013. Stata Statistical Software: Release 13. College Station,TX: StataCorp LP). The mean post value of the studied outcomes was extracted from each study. Using the forest plots, the 95% confidence interval of the difference of treatments in each study and the pooled effect of all studies were demonstrated. Heterogeneity of studies was evaluated using Cochran's Q-test and I-square index. An I-square more than 0.70 or a P-value < 0.05 was considered as the indication of heterogeneity. To compensate for the heterogeneity of the results of the studies, the Random-effects model was applied. The funnel plot (qualitative method) and Egger's regression test (quantitative method) were used for the evaluation of possible publication biases. Whenever the bias was present, the pooled mean value was adjusted using the trimming method.

##  RESULTS

The combined searches yielded 681 studies which decreased to 410 articles after the duplicates were removed. The panel reviewed 131 articles in full text based on the inclusion criteria. Of these, 36 articles (CRVO: 21 and BRVO: 15), 27 level I- or II-rated studies were selected for systematic review based on the compatibility with the inclusion criteria [Tables 1 and 2].

**Table 1:** Studies on treatment of central retinal vein occlusion associated with macular edema


**Treatment**	**Author, year (study)**	**Level**	**No. of patients**	**F/U (m)**	**Intervention, regimen**	**VA findings**	**OCT findings**	**Adverse effects**

IVT	Ip et al, 2009 (SCORE)${}^{\left[18\right]}$	I	271	12	IVT 1 mg, 4 mg, and sham groups; treated at four-month intervals as needed (49% in 1 mg IVT group and 32% in 4 mg IVT group received three doses of triamcinolone injections)	*15-letter gain: 7%, 27%, and 26% in observation, 1 mg and 4 mg groups, respectively	*At 4 months: median decrease in CMT was greater in the 4 mg triamcinolone group (–196 μm versus –77 μm in the IVT groups and –125 μm in the placebo group) *At 12 months: no difference in CMT changes	*Highest rate of cataract formation, and IOP elevation was observed in the 4 mg group.
	Ramezani et al, 2014 ${}^{\left[21\right]}$	II	86	6	IVB 1.25 mg versus IVT 2 mg; IVB group received three monthly injections of 1.25 mg of IVB, and IVT group received two injections of 2 mg IVT two months apart	*The mean BCVA: improved from 0.87 ± 0.49 to 0.41 ± 0.35 logMAR in IVB group and from 0.81 ± 0.45 to 0.62 ± 0.48 logMAR in IVT group *Inter-group differences: reached a significant level at months 4 and 6 in favor of the IVB group	*At six months: higher CMT reduction in IVB group than IVT group	*The mean IOP rise was significantly higher in the IVT group at all visits
	Ding et al, 2011${}^{\left[22\right]}$	II	32	9	IVB 1.25 mg versus IVT 4 mg; After baseline injection, patients were given additional injections if they had ME as determined by optical coherence tomography three months after the first treatment or VA loss of at least two lines	*After two weeks: the mean BCVA improvement was more in IVT compared with IVB (0.29 versus 0.35 logMAR) *After nine months: the mean BCVA improvement was maintained higher in IVT group (0.32 versus 0.38 logMAR)	*The mean CMT changes were not different between the two treatment groups at any time during the follow-up period	*Significant IOP increase was found only in the IVT group *Pre-macular membranes were developed in two patients in the IVT group
Dexa implant	Haller et al, 2011 (GENEVA)${}^{\left[23-24\right]}$	I	1,267 (437 patients with CRVO)	12	Intravitreal DEX implant 0.35 mg, 0.7 mg, and sham groups in CRVO or BRVO; After baseline injection, Intravitreal DEX implant 0.7 mg reinjection in each group at month 6	*15-letter gain at one month: 7%, 20%, and 21% in observation, 0.35 mg and 0.7 mg DEX groups, respectively *15-letter gain at two months: 9%, 23%, and 29% in observation, 0.35 mg and 0.7 mg DEX groups, respectively *15-letter gain at six months: 12%, 17%, and 18% in observation, 0.35 mg and 0.7 mg DEX groups, respectively *15-letter gain after reinjection (at 12 month): 32% of eyes in DEX 0.7 mg/0.7 mg group	Overall OCT changes (CRVO and BRVO): *after three months: significant mean decrease of –208 μm, –177 μm, and –85 μm in 0.7 mg, 0.35 mg, and sham, respectively *after six months: no differences between treatment groups *after 12 months: mean decrease of –263 μm in both 0.35 mg/0.7 mg and 0.7 mg/0.7 mg groups, while mean decrease of –267 μm in sham/0.7 mg group	* During the initial 6 months of treatment, a single intravitreal dexamethasone injection resulted in ocular hypertension in 3.9% of treated eyes compared to 0.7% in the sham group. *There were no significant differences in cataract formation at 6 months *32.8% of study eyes in 0.7 mg/0.7 mg DEX group had an IOP increase of 10 mmHg at 60 days after re-injection, which normalized at 180 days, sometimes with IOP medication.
	Hoerauf et al, 2016 (COMRADE)${}^{[}{}^{26]}$	II	185	6	IVR 0.5 mg versus intravitreal DEX implant 0.7 mg; IVR group received three monthly IVR 0.5 mg followed by PRN IVR, and the other group underwent DEX 0.7 mg implant followed by PRN sham injection	*At month 2: no difference in BCVA between IVR and DEX implant *From months 3 to 6: significant difference in BCVA gains in favor of IVR *At month 6: higher mean BCVA gain in IVR compared to DEX implant (12.86 versus 2.96 letters)	*The reduction in CMT was observed at month 2 and maintained until the end of the study in the IVR group, while the mean CMT increased in the DEX implant group starting at month 3 *after six months: mean change from baseline was –376 μm in IVR and –168 μm in DEX implant	* Elevated IOP was reported in 7 patients in the IVR group (5.6%) and 38 patients in the DEX implant group (31.9%). * Cataract occurred in 1 patient in the DEX implant group (0.8%) and no patients in the IVR group.
	Gado et al, 2014 ${}^{\left[27\right]}$	II	60	6	IVB 1.25 mg versus intravitreal DEX implant 0.7 mg; IVB group received IVB at baseline followed by PRN re-injection and the other group underwent DEX 0.7 mg implant	*After six months: no significant difference in BCVA improvement between the two groups (0.2 logMAR in each group)	*After one month: statistically significant thinner CMT in IVB group *For the rest of the six months: no significant difference between two groups	*There was a statistically significant higher IOP in DEX implant group (compared with IVB) at three–six months
	King et al, 2010 (ROCC study)${}^{\left[28\right]}$	I	32	6	IVR 0.50 mg versus sham groups; Patients received monthly IVR or sham injections for three consecutive months and then PRN re-injection with the same drug	*At three months: the BCVA improved by 16 letters in the IVR group, compared with a mean loss of 5 letters in the sham group *At six months: the BCVA improved by 12 letters in the IVR group compared with a mean loss of 1 letter in the sham group	*At three months: the mean change in CMT was –411 μm in the IVR group and 86 μm in sham *At six months: the mean change in CMT was 304 μm in the IVR and 151 μm in sham	*Two patients in the IVR group experienced a small hemorrhage in the vitreous cavity attributable to vitreous traction, which resolved without further complications
Ranibizumab	Brown et al, 2010 (CRUISE)${}^{\left[29\right]}$	I	392	6	IVR 0.3 mg, 0.5 mg, and sham groups; IVR group: received monthly intraocular injections of 0.3 or 0.5 mg of ranibizumab Sham group: received sham injection monthly	*15-letter gain: 17%, 46%, and 47% in sham, 0.3 mg and 0.5 mg IVR groups, respectively *The VA improvement after six months: the mean of 12.7 and 14.9 letters in patients receiving 0.3 mg and 0.5 mg IVR, respectively, compared with 0.8 letters in the sham group	*At six months: the mean decrease of –168 μm, –434 μm, and –452 μm in sham, 0.3 mg and 0.5 mg IVR groups, respectively	*one nonfatal myocardial infarction occurred in each treatment groups
	Campochiaro et al, 2011 (CRUISE)${}^{\left[30\right]}$	I	392	12	IVR 0.5 mg PRN re-injection after the initial six-month study in each group	*15-letter gain: 50.8% in 0.5 mg/0.5 mg group versus 33.1% in sham/0.5 mg group *The VA improvement after 12 months: the mean of 13.9 letters in both IVR groups versus 7.3 letters in sham/0.5 mg group	*At 12 months: the mean decrease of –472 μm, –453 μm, and –461 μm in sham/0.5 mg, 0.3 mg/0.5 mg, and 0.5 mg/0.5 mg respectively	*Incidence of cataract: 3.8% (0.3 mg group, 12-mo rate), 7.0% (0.5 mg group, 12-mo rate), 0% (sham; 6-mo rate)
	Heier et al, 2012${}^{\left[31\right]}$ (Horizon)	II	304 (open-label CRUISE extension)	12	IVR 0.5 mg at least every three months after the initial 12-month study in CRUISE study	*The VA improvement after 24 months from baseline: the mean of 9.4, 14.9, and 16.2 letters in the sham/0.5 mg, 0.3/0.5 mg, and 0.5/0.5 mg groups, respectively. *The BCVA worsened over the second year compared with the VA on the completion of the CRUISE study	*At 24 months from CRUISE baseline: the mean reduction was –370 μm and –412 μm in the 0.3/0.5-mg and 0.5-mg treatment groups and –418 μm in the sham/0.5-mg group *At 12 months from HORIZON baseline: the mean CFT increased by 79 μm, 88 μm, and 68 μm in the sham/0.5-mg, 0.3/0.5-mg, and 0.5-mg treatment groups, respectively	*No serious ocular and non-ocular side effect was reported
Bevacizumab	Epstein et al, 2012${}^{\left[36,37\right]}$	I	60	12	IVB 1.25 mg versus sham groups; Patients received IVB every six weeks or sham for six months, and then all patients received IVB every six weeks for second six months	*15-letter gain after six months: 60% of IVB patients versus 20% of sham patients *15-letter gain after 12 months: 60% of IVB/IVB patients versus 33% of sham/IVB patients	* After six months: the mean decrease of –426 μm versus –102 μm in IVB and sham groups * After 12 months: the mean decrease of –435 μm in IVB/IVB group versus –404 μm in sham/IVB (no difference)	*No serious ocular and non-ocular side effects was reported
	Rajagopal et al, 2015 (CRAVE)${}^{\left[38\right]}$	II	98	6	IVB 1.25 mg versus IVR 0.5 mg groups; Patients underwent monthly injection in each group	*The VA gain was 0.33 and 0.34 logMAR in IVB and IVR groups, respectively	*The CMT reduction was –212 μm and –243 μm in IVB and IVR groups, respectively	
Aflibercept	Boyer et al, 2012 (COPERNICUS)${}^{\left[43\right]}$	I	189	6	IAI 2 mg versus Sham groups; Patients underwent monthly injection in each group	*15-letter gain after six months: 56.1% of IAI patients versus 12.3% of the sham group	*After six months: the mean decrease of –457 μm versus –145 μm in IAI versus sham groups	*One case of artery occlusion and one case of maculopathy were reported in the aflibercept group
	Brown et al, 2013 (COPERNICUS)${}^{\left[44\right]}$	I	189	12	Extension of Boyer et al study; After six months, both IAI and sham groups continued to receive aflibercept for the next six months, PRN	*15-letter gain after 12 months: 55.3% of IAI/IAI group versus 30.1% of sham/IAI group	*After 12 months: the mean decrease of –413 μm and –381 μm in IAI/IAI and sham/IAI groups, respectively	*No intraocular or extra ocular complication
	Heier et al, 2014 (COPERNICUS)${}^{\left[45\right]}$	I	188	24	Extension of Brown et al study; After 12 months, patients were evaluated at least quarterly and received IAI PRN	*15-letter gain after 24 months: 49.1% in IAIQ4weeks/IAI PRN group versus 23.3% in Sham/IAI PRN group *The mean VA gain after 24 months: 13 versus 1.5 letters in each group, respectively	*After 24 months: the mean CMT was reduced –390 μm and –343 μm in IAIQ4weeks/IAI PRN group versus Sham/IAI PRN group, respectively	*The most frequent ocular serious adverse event from baseline to the month 24 was vitreous hemorrhage in both groups
	Holz et al, 2013 (GALILEO)${}^{\left[46\right]}$	I	177	6	IAI 2 mg versus sham groups; Patients underwent monthly injection in each group	*15-letter gain after six months: 60% in IAI group versus 22% in the sham group *The mean gain VA after six months: a mean of 18 letters in IAI compared to 3.3 letters in the sham group	*After six months: the mean decrease of –449 μm versus –169 μm in IAI and sham groups, respectively	*No intraocular or extra ocular complication
	Korobelnik et al, 2014 (GALILEO)${}^{\left[47\right]}$	I	177	12	Extension of Holz et al study; From month 7 to 12, the IAI group received aflibercept PRN and the sham group continued receiving sham injections	*15-letter gain after 12 months: 60.2% in the IAI group and 32.4% in the sham group *The mean gain VA after 12 months: a mean of +16.9 letters versus +3.8 letters in IAI and sham groups, respectively	*After 12 months: the mean CMT reduction from baseline was –432 μm versus –219 μm in IAI and sham groups, respectively	*Increased intraocular pressure was reported in 17.3% of injections that resolved spontaneously
	Ogura et al, 2014 (GALILEO)${}^{\left[48\right]}$	I	177	18	Extension of Korobelnik et al study; From months 13 to 18, patients were monitored every eight weeks, and both groups received IAI 2 mg PRN	*15-letter gain after 18 months: 57.3% in the IAI/IA group and 29.4% in the sham/IAI group *The mean gain VA after 18 months: a mean of +13.7 letters versus +6.2letters in IAI/IAI and sham/IAI groups, respectively	*After 18 months: the mean CMT reduction from baseline was –389 μm versus –306 μm in IAI/IAI and sham/IAI groups, respectively	*No intraocular or extra ocular complication
	Saishin et al, 2017${}^{\left[49\right]}$	II	26	6	IVR 0.5 mg versus IAI 2 mg groups; Both groups received bimonthly injections	*The BCVA improvement after six months: 0.31 logMAR versus 0.20 logMAR in IVR and IAI groups, respectively (not significant)	*After six months: the mean CMT reduction from baseline was –374 μm versus –465 μm in IVR and IAI groups, respectively (the difference was not statistically significant)	*No serious complication was reported
	Scott et al, 2017 (SCORE2)${}^{\left[50\right]}$	I	362	6	IAI 2 mg versus IVB 1.25 mg groups;newline Both groups received monthly injections	*The BCVA improvement after six months: 19 letter versus 18.9 letter in IAI and IVB groups, respectively (not significant)*15-letter gain after six months: 65.1% in the IAI group compared with 61.3% in the IVB group	*After six months: the mean CMT reduction from baseline was –425 μm versus –387 μm in IAI and IVB groups, respectively (the difference was not statistically significant)	*In the IAI group: four participants with IOP more than 10 mm Hg greater than baseline; *In the IVB group: nine participants with IOP more than 10 mm Hg greater than baseline and two patients with IOP higher than 35 mmHg were reported
	Casselholm De Salles et al, 2018${}^{\left[51\right]}$	II	45	18	IAI 2 mg versus IVR 0.5 mg groups; After three loading doses, the treatment intervals were extended by 2 weeks to a maximum of 12 weeks. Intervals were shortened by two weeks if edema recurred	*The BCVA improvement after 18 months: 22.4 letter versus 20 letter in IAI and IVR groups, respectively (not significant) *15-letter gain after 18 months: 67.4% in the whole cohort	*After 18 months: the mean CMT reduction from baseline was –550.4 μm versus –551.8 μm in IAI and IVR groups, respectively (the difference was not statistically significant)	*No intraocular complication was reported
	
	
CMT, central macular thickness; DEX, dexamethasone; F/U, follow up; IAI, intravitreal aflibercept injection; IOP, intraocular pressure; IVB, intravitreal bevacizumab; IVR, intravitreal ranibizumab; IVT, intravitreal triamcinolone; ME, macular edema; OCT, optical coherent tomography; VA, visual acuity								

**Table 2:** Studies on the treatment of branch retinal vein occlusion associated with macular edema


**Treatment**	**Author, year (study)**	**Level**	**No. of patients**	**F/U (m)**	**Intervention, regimen**	**VA findings**	**OCT findings**	**Adverse effects**

IVT	Scott et al, 2009 (SCORE)${}^{[18}{}^{-}{}^{54]}$	I	411	12	IVT 1 mg, 4 mg, and grid laser photocoagulation groups; Treated at four-month intervals as needed	*15-letter gain: 28.9%, 27.2%, and 25.6% in laser, 1 mg and 4 mg groups, respectively *After 12 months: -IVTA 4 mg:.4.0 letters gain, -IVTA 1 mg:.5.7 letters gain; -Laser: 4.2 letters gain	*At four months: -IVTA 4 mg: –142 μm -IVTA 1 mg: –77 μm -Laser group: –113 μm *CMT in the 4 mg triamcinolone group had decreased significantly compared to the other groups *At 12 months: -IVTA 4 mg: –170 μm -IVTA 1 mg: –149 μm -Laser group: –224 μm *At the 12-month follow-up, all groups had experienced a similar mean reduction in CMT	*Highest rate of cataract formation and IOP elevation was observed in the 4 mg group
Dexa implant	Haller et al, 2011 (GENEVA) ${}^{[23}{}^{,}{}^{24}{}^{]}$	I	1,267 (830 patients with BRVO)	12	Intravitreal DEX implant 0.35 mg, 0.7 mg, and sham groups in CRVO or BRVO; After baseline injection, Intravitreal DEX implant 0.7 mg reinjection in each group at month 6	*15-letter gain at one month: 8%, 17%, and 21% in observation, 0.35 mg and 0.7 mg DEX groups, respectively *15-letter gain at two months: 15%, 23%, and 24% in observation, 0.35 mg and 0.7 mg DEX groups, respectively *15-letter gain at six months: 20%, 21%, and 23% in observation, 0.35 mg and 0.7 mg DEX groups, respectively *15-letter gain after reinjection (at 12 months): 32% of eyes in DEX 0.7 mg/0.7 mg group (CRVO + BRVO)	Overall OCT changes (CRVO and BRVO): *After three months: significant mean decrease of –208 μm, –177 μm, and –85 μm in 0.7 mg, 0.35 mg, and sham, respectively *After six months: no differences between treatment groups *After 12 months: mean decrease of –263 μm in both 0.35 mg/0.7 mg and 0.7 mg/0.7 mg groups, while mean decrease of –267 μm in sham/0.7 mg group	*32.8% of study eyes in 0.7 mg/0.7 mg DEX group had an IOP increase of 10 mmHg at 60 days, which normalized at 180 days, sometimes with IOP medication (CRVO and BRVO)
	Guignier et al, 2013${}^{\left[55\right]}$	II	19	6	IVB 1.25 mg versus intravitreal DEX implant 0.7 mg; IVB group received three monthly IVB 1.25 mg followed by PRN IVB injection, and the other group underwent DEX 0.7 mg implant followed by PRN DEX re-injection in the fourth month	*At month 1: the mean VA was significantly better in dexamethasone group *Months 2 to 6: there was no longer any difference between the two groups *Although there was no difference in mean VA values between the two groups at six months, the proportion of eyes that gained 15 ETDRS letters or more was higher in IVB group than in DEX implant group (30% versus 11%)	*At month1: the mean CMT was significantly lower in the dexamethasone group *Months 2 to 6: there was no longer any difference between the two groups	*Elevated IOP was reported in 9% of patients in the dexamethasone group while there was none in IVB group
Ranibizumab	Campochiaro et al, 2010 (BRAVO)${}^{\left[56\right]}$	I	397	6	IVR 0.3 mg, 0.5 mg, and sham groups; IVR group received monthly intraocular injections of 0.3 or 0.5 mg of ranibizumab for six months	*15-letter gain: 28.8%, 55.2%, and 61.1% in sham, 0.3 mg and 0.5 mg IVR groups, respectively. *The VA improvement after six months: the mean of 16.6 and 18.3 letters in patients receiving 0.3 mg and 0.5 mg IVR respectively, compared with 7.3 letters in the sham group	*At six months: the mean decrease of –157 μm, –337 μm, and –345 μm in sham, 0.3 mg, and 0.5 mg IVR groups, respectively	*One case of endophthalmitis in IVR 0.5 mg group
	Brown et al, 2011 (BRAVO)${}^{\left[57\right]}$	I	397	12	IVR 0.5 mg PRN injection after the initial six-month study in sham group The 0.3 mg and 0.5 mg groups were continued with their original doses	*15-letter gain: 56% and 60.3% in 0.3 mg and 0.5 mg group versus 43.9% in sham/0.5 mg group *The VA improvement after 12 months: the mean of 16.4 and 18.3 letters in 0.3 mg and 0.5 mg groups versus 12.1 letters in sham/0.5 mg group	*At 12 months: the mean decrease of –273 μm, –313 μm, and –347 μm in sham/0.5 mg, 0.3 mg, and 0.5 mg, respectively *The mean improvement from baseline CMT at month 12 in the sham/0.5 mg group was significantly less than that of the other treatment groups	*Cataract rate: 4.5% [0.3 mg], 6.2% [0.5mg], and 3.1% [sham/0.5 mg] *Vitreous hemorrhage: 5.2% [0.3 mg], 1.5% [0.5 mg], and 4.6% [sham/0.5 mg]
	Heier et al, 2012 (Horizon)${}^{\left[31\right]}$	II	304 (open-label BRAVO extension)	12	IVR 0.5 mg at least every three months after the initial 12-month study in BRAVO study	*The VA improvement after 24 months from baseline: the mean of 15.6, 14.9, and 17.5 letters in the sham/0.5 mg, 0.3/0.5 mg, and 0.5/0.5 mg groups, respectively *The BCVA worsened over the second year compared with the BCVA on the completion of the BRAVO study except for the sham/0.5 mg group. -IVR 0.3 mg/0.5 mg: –2.3 -IVR 0.5 mg/0.5 mg: –0. 7 -Sham/0.5 mg: +0.9	*At 24 months from BRAVO baseline: the mean reduction was –307 μm and –360 μm in the 0.3/0.5-mg and 0.5-mg treatment groups and –298 μm in the sham/0.5-mg group *At 12 months from HORIZON baseline: the mean CMT increased by +3.7 μm, +6.3 μm, and +35.3 μm in the sham/0.5-mg, 0.3/0.5-mg, and 0.5-mg treatment groups, respectively	*Increased IOP in two patients all over the groups *No non-ocular side effect was reported
	Tadayoni et al, 2016 (BRIGHTER)${}^{\left[58\right]}$	I	455	6	IVR 0.50 mg, IVR 0.5 mg plus laser photocoagulation, and laser photocoagulation alone; Patients treated with ranibizumab with or without laser received a minimum of three initial monthly ranibizumab injections until VA stabilization, and then VA-based PRN dosing	*15-letter gain after six months was 47.2%, 45%, and 27.8% in IVR plus laser, IVR alone, and laser alone groups, respectively*At six months: The BCVA improved by 14.8 letters in the IVR with and without laser groups, compared with a mean gain of six letters in the laser group	*At six months: the mean change in CMT was –240 μm in the IVR group, –223 μm in IVB plus laser group, and –87 μm in the laser group	*Conjunctival hemorrhage and eye pain were the most commonly reported ocular adverse events in all groups
	Tan et al, 2014${}^{\left[59\right]}$	II	36	12	IVR 0.50 mg versus sham; IVR group received monthly intraocular injections of 0.5 mg of ranibizumab for six months and then PRN Sham group received monthly sham injections Grid laser was administered at 13 and 25 weeks in both groups if criteria for laser treatment were met	*The VA improvement after 12 months in IVR group: +12.5 letter *The VA worsened in the sham group (–1.6 letter) despite rescue laser	*At 12 months: the mean change in CMT was –361 μm in the IVR group and –175 μm in the sham group	*No new ocular or systemic adverse events were observed
	Pielen et al, 2015 (RABAMES)${}^{\left[60\right]}$	II	31	6	IVR 0.50 mg, grid laser, or both; Patients with a BCVA between 20/320 and 20/40 were randomized 1:1:1 to receive grid laser or three monthly injections of 0.5 mg IVR or both followed by three months of observation	*15-letter gain after six months was 70% in both IVR and IVR + laser group versus 20% in the laser group *Mean change from baseline BCVA at month 6 was +2, +17, and +6 letters in laser, IVR, and combination therapy, respectively	*At month 6: the mean decrease in CMT was –128 μm in the laser group, –237 μm in the ranibizumab group, and –97 μm in the combination group (*p* = 0.10 for IVR versus laser group and *p* = 0.08 for IVR versus combination group)	*No serious ocular adverse event *One case of stroke after the first intravitreal injection of ranibizumab which resulted in study discontinuation according to the patient's decision
Bevacizumab	Parodi et al, 2015${}^{\left[64\right]}$	II	35	12	IVB 1.25 mg versus subthreshold laser groups; IVB 1.25 mg was given at baseline and then on a PRN regimen according to ME presence on OCT. The subthreshold laser was administered once	*15-letter gain after 12 months: 58% in the bevacizumab group versus no change in BCVA in laser group *The mean BCVA changed from 0.92 ± 0.3 (LogMAR) to 0.99 ± 0.2 in the laser group, while in the IVB group, the mean BCVA showed a statistically significant improvement from 0.94 ± 0.3 to 0.72 ± 0.2	*After 12 months: CMT was significantly improved in the bevacizumab group (–213 μm) and was unchanged in the laser group	*No serious ocular and the non-ocular side effect was reported
	Narayanan et al, 2015 (MARVEL)${}^{\left[65\right]}$	II	75	6	IVB 1.25 mg versus IVR 0.5 mg; Eyes were treated with IVB or IVR injection at baseline followed by monthly PRN re-injections	*15-letter gain after six months: 59.4% and 57.8% in IVR and IVB groups, respectively *The BCVA improvement from baseline: 18.1 versus 15.6 in IVR and IVB groups, respectively	*The mean CMT decreased –177 μm versus –201 μm in IVR and IVB groups, respectively	*No serious ocular and non-ocular side effect
	Cekic et al, 2010${}^{\left[66\right]}$	II	31	6	IVB 1.25 mg versus IVTA 4 mg	*The BCVA improvement from baseline: 7 letters versus 24 letters in IVTA and IVB groups, respectively	*The CMT reduction was –190 μm and –132 μm in IVTA and IVB groups, respectively	*Compared to baseline, average intraocular pressure change from baseline was significantly higher in IVTA group at one month after injection while those of IVB group were not different
	Higashyama et al, 2010${}^{\left[67\right]}$	I	43	12	IVB 1.25 mg versus IVTA 4 mg; Single injection at baseline with additional treatments allowed as needed after three months	*The BCVA improvement from baseline: 16.5 letters versus 11 letters in IVB and IVTA groups, respectively	*The CMT reduction was –262 μm and –304 μm in IVB and IVTA groups, respectively	*Elevated IOP: -IVB: 11% -IVTA: 22%
Aflibercept	Campochiaro et al, 2015 (VIBRANT)${}^{\left[68\right]}$	I	183	6	IAI 2 mg versus Laser; Treating monthly with IAI 2 mg in IAI group; the laser was done at baseline and then repeated at 12 weeks	*15-letter gain after six months: 52.7% of IAI patients versus 26.7% of the laser group *The BCVA improvement from baseline: 17 versus 6.9 letters in IAI and laser groups, respectively	*After six months: the mean decrease in CMT was –280 μm versus –128 μm in IAI versus laser groups, respectively	*Common adverse events: -Subconjunctival hemorrhage IAI: 19.8% Laser: 4.3% -Retinal neovascularization IAI: 0% Laser: 3%
	Clark et al, 2016 (VIBRANT)${}^{\left[69\right]}$	I	183	12	After six months, IAI group continued to receive aflibercept every eight weeks (instead of every four weeks) for the next six months. The laser-only group was allowed to receive IAI every eight weeks	*15-letter gain after 12 months: 57.1% of IAI/IAI group versus 41.1% of laser/IAI group *The BCVA improvement from baseline: 17.1 versus 12.2 letters in IAI/IAI and laser/IAI groups, respectively	*After 12 months: the mean decrease of –283 μm and –249 μm in IAI/IAI and laser/IAI groups, respectively	*Traumatic cataract in one eye (1.1%) in the IAI group was the only serious ocular adverse event
	
	
CMT, central macular thickness; DEX, dexamethasone; F/U, follow up; IAI, intravitreal aflibercept injection; IOP, intraocular pressure; IVB, intravitreal bevacizumab; IVR, intravitreal ranibizumab; IVT, intravitreal triamcinolone; ME, macular edema; OCT, optical coherent tomography; PRN, pro re nata; VA, visual acuity								

**Figure 1:**
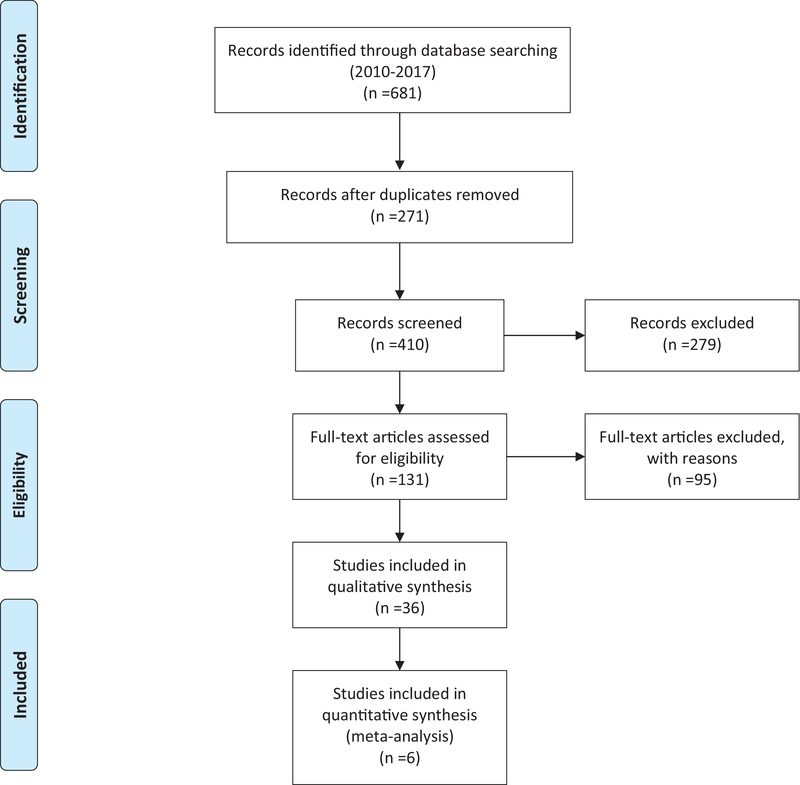
PRISMA flowchart. **Source:** Moher D, Liberati A, Tetzlaff J, Altman DG, The PRISMA Group. Preferred reporting items for systematic reviews and meta-analyses: The PRISMA Statement. PLoS Med 6(7): e1000097. For more information, visit www.prisma-statement.org.

Studies with level I evidence and comparable arms and methods were chosen for meta-analysis. Meta-analysis was done on studies that compared the effectiveness of bevacizumab versus triamcinolone acetonide and ranibizumab versus sham. Due to the heterogeneity in the dosages of intravitreal steroid and follow-up periods, we were not able to perform a meta-analysis on all RCTs; six RCTs were selected for meta-analysis based on the inclusion and exclusion criteria [Figure 1].

###  Medical Management in CRVO

#### Description of the Condition in CRVO

Macular edema is one of the main causes of reduced VA in CRVO. It is presumed to be a result of the hypoxia-induced capillary permeability after vein occlusion and subsequent hemorrhage.^[15]^ The most popular treatment in CRVO is the intravitreal injection of an anti-VEGF, such as bevacizumab, ranibizumab, and aflibercept. However, intravitreal steroid injection may also be considered especially in areas without access to anti-VEGF agents. Intravitreal dexamethasone implant is another therapeutic choice for CRVO-induced ME (CRVO-ME),^[16]^ while other treatments such as fibrinolytic or anticoagulant agents, angiostatic agents, acetazolamide and isovolemic hemodilution, have not been approved.^[17]^ Some surgical options were also suggested including induced chorioretinal anastomosis, injection of a fibrinolytic agent by direct venous cannulation, and radial optic neurotomy; however, none of them has been proven effective in the treatment of CRVO-ME.^[17]^ The summary of the interventional studies on the management of CRVO-ME with level I or II of evidence are presented in Table 1.

#### Intravitreal Corticosteroids

*Triamcinolone*. Intravitreal injection of steroids is effective in CRVO-ME by reducing the capillary permeability and inhibition of the VEGF expression. In contrast to anti-VEGF drugs with specific site of action, steroids suppress the expression of many cytokines. However, they may cause cataract and steroid-induced glaucoma. It should be noted that the effects of intravitreal triamcinolone may last up to nine months, depending on the dosage applied.^[18,19,20]^

Ip et al^[18]^ reported the results of the Standard Care versus Corticosteroid for Retinal Vein Occlusion (SCORE) study that compared 1 mg and 4 mg doses of preservative free intravitreal triamcinolone acetonide (IVT) with the observation for ME-associated VA loss in perfused CRVO. At four-month follow-up, the median reduction in CMT was more in the 4 mg IVT group (P < 0.001). However, at 12-month follow-up, there was no difference in terms of CMT reduction and VA improvement between the two triamcinolone groups. IVT led to a reduction in ME with a moderate correlation with VA. They reported that there was a significantly higher percentage of patients requiring IOP-lowering medications and also higher rates of cataract development in the steroid groups, especially in the 4 mg IVT group. Considering these efficacy and safety findings, 1 mg IVT was recommended in the treatment of CRVO-ME. In this study, the interval between the last triamcinolone application and the end of the follow-up period varied. Hence, some eyes in the study groups might have been out of the effective phase of the triamcinolone injection at the evaluation time.

Ramezani et al^[21]^ evaluated the effects of multiple intravitreal bevacizumab (IVB) injections versus IVT in the treatment of 86 eyes with ME due to acute CRVO. It was interesting that the differences were statistically significant in patients with ischemic CRVO in favor of the IVB group. At all visits, mean IOP rise was significantly higher in the IVT group in comparison with the IVB group. As a result, they recommended repeated IVB injections, specifically in ischemic cases.

Ding et al^[22]^ compared the safety and efficacy of IVB and IVT in patients with CRVO. No statistically significant difference was found during the nine-month follow-up between the two treatment groups. Quicker visual recovery and improvement of CMT was observed in patients who underwent IVT injection compared with patients who received IVB. In addition, fewer injections were needed in the IVT group (1.31 ± 0.48) in comparison with the IVB group (2.38 ± 1.04). In this study, retreatment with IVB was performed three months after the first injection if persistent ME was observed, instead of three consecutive monthly injections in the first three months. Therefore, the IVB group might have been undertreated in this study.

*Dexamethasone Intravitreal Implant (Ozurdex)*. Intravitreal dexamethasone implant is a slow-release steroid in a biodegradable polymer form that lasts about three to four months.^[23]^ The results of the Global Evaluation of implantable Dexamethasone in Retinal Vein Occlusion with Macular Edema (GENEVA) trial were reported by Haller et al.^[23]^ They evaluated the efficacy and safety of intravitreal dexamethasone implant in two dosages compared with placebo injection in eyes with RVO-ME (either BRVO or CRVO). A total of 1,267 patients with VA between 20/50 and 20/200 were enrolled. Patients with dexamethasone implant (either 0.35 mg or 0.70 mg) improved significantly faster compared to the placebo regimen. From the first month to the third month of treatment, more eyes in the dexamethasone implant group achieved 15 ETDRS letters compared to the placebo group (P < 0.001); the maximum response was observed at month 3. However, the proportion of eyes achieving 15 ETDRS letter improvement after six months was not different between the three groups. The mean VA improvement was more pronounced in the dexamethasone groups compared to the placebo group from month 1 to month 6 (P = 0.006); the greatest difference was observed at the end of the second month which was about seven ETDRS letters. The mean decrease in CMT was also marked significantly more in eyes receiving each dose of dexamethasone implant compared with placebo at month 3; however, the difference was not sustained up to month 6. It seemed that these changes in CMT were parallel to changes in BCVA. The observed clinical efficacy of dexamethasone implant had a limited duration, lasting between 90 to 120 days with a gradual decline thereafter. However, the peak response was observed after three months and was accompanied with the highest incidence of IOP rise between days 60 to 90. This study embraced a large proportion of non-ischemic CRVO patients that led to encouraging outcomes, but the use of dexamethasone in ischemic CRVO remained questionable. Visual acuity improvement was sustained after the extension of the GENEVA study to 12 months by repeated injections of dexamethasone implant. On the other hand, complications were observed more frequently with repeated doses of dexamethasone implant, but these side effects could be satisfactorily managed by either surgery or medications. After the second dexamethasone implant, a large number of patients were able to maintain the visual gain of more than 15 ETDRS letters beyond six months although more steroid-related complications might occur.^[24]^

In SOLO study (Functional and Anatomical Results after a Single Intravitreal Ozurdex Injection in Retinal Vein Occlusion), Bezatis et al evaluated the efficacy period of Ozurdex (dexamethasone implant) in both CRVO- and BRVO-related ME. Like Geneva study, this study showed that early reinjection of dexamethasone implant after 16 weeks instead of 24 weeks was required in most cases; 40.7% of the BRVO group and 15% of the CRVO group required reinjection after 17.5 and 17.6 weeks, respectively.^[25]^

Hoerauf et al in COMRADE C study (Clinical Efficacy and Safety of Ranibizumab Versus Dexamethasone for CRVO) evaluated the efficacy of dexamethasone implant versus ranibizumab in eyes with CRVO-ME.^[26]^ They showed that although both treatment groups had a similar outcome in the first two months, the efficacy of ranibizumab sustained throughout the study, while dexamethasone therapeutic effect decreased from month 3 onward. The limitation was that all eyes in the dexamethasone group were treated with only a single dosage during the six-month period of the study. Based on the GENEVA and SOLO studies, the effect of treatment gradually decreases after three months and re-injection is needed. In other words, in clinical practice, dexamethasone re-implantation might be required earlier than six months.

In a randomized clinical trial by Gado et al, the efficacy of intravitreal dexamethasone implant versus repeated bevacizumab injections were evaluated in six months. They showed that both drugs provided the same effect on VA gain and CMT reduction after the first six months of treatment although there was a significantly higher rate of IOP rise in the dexamethasone implant group compared with the IVB group at three-six months.^[27]^

#### Anti-VEGF Agents

*Ranibizumab*. ROCC study (a Randomized Study Comparing Ranibizumab to Sham in Patients with ME Secondary to CRVO) was performed to compare ranibizumab with placebo regimen in patients with CRVO-ME. At each time point, BCVA was improved in the ranibizumab group compared with the visual loss in the sham group (P = 0.001). Eighty percent of cases (n = 12) in the ranibizumab group required more than three initial injections (4.3 ± 0.9) during the study. They concluded that monthly ranibizumab injection significantly improved ME and BCVA; maintaining the initial improvement would be possible with consecutive repeated injections.^[28]^

Ranibizumab for the Treatment of Macular Edema after Central Retinal Vein Occlusion CRVO (CRUISE) trial compared the efficacy of ranibizumab 0.3 mg or 0.5 mg with placebo in CRVO-ME.^[29]^ After six months, the VA of patients treated with either 0.3 mg or 0.5 mg ranibizumab improved significantly more than the placebo group. Additionally, anatomical changes correlated well with the visual improvements. Later, Campochiaro et al^[30]^ published the 12-month results of the CRUISE trial. In this extension of the study, patients were eligible to be treated with 0.5 mg ranibizumab on a pro re nata (PRN) basis if BCVA was less than 20/40 or CMT was more than 250 μm. The superiority regarding the mean ETDRS letter gain after one year was maintained in the ranibizumab treatment groups compared with the placebo group. After 12 months, the mean reduction of CMT was similar between the study groups. The number of needed injections was of interest as it determined the financial burden. The mean number of injections among all randomized patients was 3.3 to 3.8 at months 6 to 12 based on the PRN approach. As a result, ranibizumab was superior to sham based on the visual improvement at months 6 and 12, and the 0.5 mg ranibizumab was a more effective regimen in the treatment of CRVO-ME, based on this study.

HORIZON trial (Ranibizumab for Macular Edema due to Retinal Vein Occlusions: Long-term Follow-up) provided data regarding ranibizumab use in 24 months and analyzed the long-term safety and efficacy of ranibizumab in RVO-related ME (either BRVO or CRVO).^[31]^ It was a multicenter single-armed study with 304 patients from the BRAVO and 304 patients from the CRUISE studies being recruited. They were evaluated at three-month intervals and were candidates for 0.5 mg intravitreal ranibizumab if the recurrence of ME or a drop in VA was identified. Indeed, HORIZON was an extension study of the BRAVO and CRUISE studies. In the second year, fewer ranibizumab injections in patients with CRVO was associated with a more prominent worsening of visual outcomes compared to the BRVO patients. A reasonable explanation is that retinal ischemia is more extensive in CRVO, leading to a larger VEGF drive that requires more frequent doses of ranibizumab injections. Hence, fewer ranibizumab injections and reduced follow-up in the second year of treatment resulted in a decline in VA of CRVO patients.

A post-hoc analysis study based on the data obtained by BRAVO and CRUISE trials evaluated the effects of ranibizumab on RVO-related ME (789 patients: BRAVO, n = 397; CRUISE, n = 392). VA improvement was observed just after seven days post-injection and was persistent up to 12 months by the PRN regimen. The time to first gain of 15 ETDRS letters of VA in the CRVO group was 4 months and in the BRVO group was 5.2 months after repeated 0.5 mg monthly ranibizumab injections. Overall, more than half of the patients who received monthly regimen achieved significant functional improvement after the first six months of the treatment.^[32]^

*Bevacizumab*. Several retrospective and prospective studies reported the efficacy of intravitreal injection of bevacizumab in the improvement of VA and reduction of CMT.^[22,33,34,35]^ In a prospective controlled clinical trial by Epstein et al,^[36]^ after six months, three times more patients in the study group had gained at least 15 letters than the sham group. The mean CMT reduction was significantly more pronounced in the bevacizumab group compared to the control group. The authors continued the study with a six-month extension period.^[37]^ From 6 months to 12 months, all patients from both groups were candidates to be treated with PRN bevacizumab every six weeks. CMT reduction was prominent in both sham/bevacizumab and bevacizumab/bevacizumab groups. However, vision improvement was more prominent in the bevacizumab/bevacizumab group versus the sham/bevacizumab group. Although anatomic improvement occurred after crossing over of sham patients to bevacizumab, functional improvement was limited. Therefore, earlier treatment might lead to better outcomes.

Rajacopare et al in CRAVE study (Bevacizumab Versus Ranibizumab in the Treatment of Macular Edema Due to Retinal Vein Occlusion)^[38]^ compared the efficacy of monthly ranibizumab or bevacizumab for RVO-ME in a randomized clinical trial. After six months, changes in CMT and VA were not different between the treatment groups. Although the efficacy of bevacizumab and ranibizumab are reported to be quite similar in the treatment of CRVO-ME in many studies,^[39,40,41]^ the use of bevacizumab in the management of RVO-ME remains off-label.

Ding et al^[22]^ evaluated the safety and efficacy of IVT versus IVB for CRVO-ME. After nine months, 5 of the 16 IVT eyes and 12 of the 16 IVB eyes needed re-treatment. The mean number of injections in the triamcinolone group (1.3) was less than in the bevacizumab group (2.4). BCVA improvement occurred at all time points after the injections in both study groups, and no significant difference was observed between the two groups.

Some non-randomized studies reported more chance for vision gain in eyes treated with a combination of dexamethasone and IVB than eyes that received dexamethasone implant monotherapy^[42]^.

*Aflibercept*. The protein VEGF Trap-Eye (aflibercept) comprises key domains of human VEGF receptors 1 and 2, fused with human IgG FC fragment. Indeed, all isoforms of VEGF-A and placental growth factor would be blocked by this protein. The main advantage of aflibercept is its longer duration of activity.^[43]^ Hence, it may reduce the dosing intervals of intravitreal injections in comparison with ranibizumab and bevacizumab. GALILEO (General Assessment Limiting Infiltration of Exudates in Central Retinal Vein Occlusion with VEGF Trap-Eye) and COPERNICUS (Vascular Endothelial Growth Factor [VEGF] Trap-Eye: Investigation of Efficacy and Safety in Central Retinal Vein Occlusion) are multicenter randomized clinical studies that evaluated the intravitreal aflibercept effects in patients with CRVO-ME.^[43,44,45,46,47,48]^

In COPERNICUS study,^[43]^ VA improvement and macular thickness reduction after six months were significantly more in the aflibercept treated eyes than the control group. They also reported that reduced ocular neovascularization was noted in the aflibercept group. Later, Brown et al^[44]^ reported the 12-month results of COPERNICUS study in which the previously established arms for the RCT were eligible to be treated with PRN doses of aflibercept 2 mg every four weeks from month 7 to month 12. After 12 months, the mean VA improvement was 16.2 and 3.8 letters in the aflibercept + PRN group and the sham + PRN group, respectively. They concluded that fewer injections of aflibercept after the loading period could maintain the VA gain; however, a delay in the treatment of ME could lead to irreversible damage due to chronic edema and disintegrated retinal layers. Heier et al^[45]^ reported the 24-month results of the study. After the first year, based on the study protocol, patients were evaluated every three months and were treated with aflibercept if needed. The visual gain was significantly more in the aflibercept/PRN group than the sham/PRN group after two years (13 versus 1.5 letters in each group, respectively). The mean number of PRN injections was less in the aflibercept + PRN (2.7 ± 1.7) compared with the sham + PRN (3.9 ± 2.0) during months 7 to 12, while during months 13 to 24 the number of PRN injections were 3.3 ± 2.1 versus 2.9 ± 2.0 in the two groups, respectively. They also showed that the functional and anatomic improvements, after fixed dosing through six months, followed by PRN regimen and monthly monitoring from months 7 to 12, were reduced after continued PRN regimen and a reduced monitoring frequency from months 12 to 24 [Table 1]. They suggested a beneficial effect of anti-VEGF treatment in either group of patients with ischemic or non-ischemic CRVO; this effect was significantly lower if treatment was started with a delay in both subgroups. Considering the time between diagnosis and treatment, they reported that the proportion of patients with 15 letter gain or more after six months was significantly more in patients who received the first intravitreal aflibercept injection during the first two months of diagnosis. They also showed that aflibercept is beneficial even in patients with poor-presenting VA of less than 20/200.

In a similar double masked RCT named GALILEO, patients with CRVO were randomized to intravitreal aflibercept 2 mg or placebo monthly for six months. Based on GALILEO reports, the mean change in BCVA and CMT were more marked in patients receiving aflibercept than the sham group at week 24 (P = 0.0001).^[46]^ From months 7 to 12, patients were monitored monthly; the aflibercept group was treated with the intravitreal drug on a PRN basis, and the placebo group continued to receive sham. From months 13 to 18, patients were monitored bimonthly, and both groups were treated with intravitreal aflibercept PRN. This study showed that the functional and anatomic improvements after fixed monthly dosing in the first six months were largely sustained even with the extension of the treatment intervals. Patients with a baseline BCVA of 20/200 or worse had a greater visual improvement after 12 months compared to patients with a baseline BCVA of better than 20/200. Similar to COPERNICUS study, this study revealed that, although delayed treatment with aflibercept led to anatomic improvement, the functional improvement was limited, and the effect could persist for two years. It showed that more improvement of vision might occur if treatment was started earlier.^[47,48]^

Based on these studies, significant visual and anatomic improvements occurred in the first six months with fixed monthly aflibercept injections. These improvements were largely maintained by PRN aflibercept injection with a mean of 2.5 to 2.7 injections in the next six months. Therefore, a monthly loading dose for up to three months followed by bimonthly aflibercept may have similar efficacy to that of monthly ranibizumab.^[47,48]^

The efficacy of bimonthly intravitreal aflibercept and ranibizumab in CRVO-ME was evaluated by Saishin et al. They concluded that although no significant difference was observed in visual improvement between the two groups, VEGF may not be completely neutralized by bimonthly injections of ranibizumab in all patients with CRVO, which may lead to the recurrence of ME.^[49]^

Recently, in SCORE II study, the efficacy of monthly bevacizumab was compared with monthly aflibercept in patients with CRVO-ME. Based on this study, monthly bevacizumab was not inferior to aflibercept regarding visual improvement after six months. SCORE II extension studies probably will compare outcomes of these anti-VEGF agents after six months, using regimens other than monthly dosing.^[50]^

In a recent report of a randomized clinical trial, the injection frequency of aflibercept and ranibizumab in the treatment of CRVO-ME was investigated. Patients were allocated to receive either intravitreal aflibercept (2mg) or ranibizumab (0.5mg) in a treat and extent regimen. After 18 months, the number of injections was significantly lower in the aflibercept group (10.9) compared to the ranibizumab group (14.4) (p = 0.001). The mean treatment interval was significantly longer in the aflibercept group compared with the ranibizumab group (10.0 versus 6.6 weeks) (p = 0.001). The mean changes in BCVA and CMT were similar between the groups. The authors concluded that the application of treat and extent regimen with aflibercept in eyes with CRVO might reduce the treatment burden and, to some extent, the need for close monitoring of patients.^[51]^

###  Medical Management of BRVO

Similar to CRVO, ME is the main reason for visual loss in BRVO. Based on the Branch Vein Occlusion Study (BVOS), macular grid laser photocoagulation was an effective treatment in branch retinal vein occlusion-related macular edema (BRVO-ME).^[9]^ Pharmacologic intervention started with the use of corticosteroids: triamcinolone and dexamethasone. Multiple formulations of IVT were used as an off-label drug in the treatment of BRVO-ME. The intravitreal dexamethasone sustained-release implant was approved in 2009 for the treatment of BRVO-ME by the U.S. Food and Drug Administration (FDA).^[23,24]^

In recent years, anti-VEGF agents (ranibizumab, aflibercept, and bevacizumab) have become the most popular therapeutics for BRVO-ME. In addition, other modalities have been evaluated in the treatment of BRVO including chorioretinal anastomosis with laser, separation of the common adventitia of the crossing artery and vein with removing the cortical vitreous following pars plana vitrectomy, and intra-vascular injection of t-PA through cannulation of the veins.^[52,53]^ Interventional studies on the management of BRVO-ME with levels I or II of evidence are summarized in Table 2.

####  Intravitreal Corticosteroids

*Triamcinolone*. In a study designed for the treatment of BRVO-ME, Scott et al compared the effectiveness of standard care (grid laser photocoagulation) versus corticosteroid for retinal vein occlusion (SCORE) study report 6.^[54]^ This study was conducted to evaluate the efficacy of two doses of preservative-free intravitreal triamcinolone with grid photocoagulation in BRVO-ME patients with BCVA between 20/40 and 20/400 and CMT of more than 250 μm. At month 12, the VA improvement was similar between the groups; however, more complications were observed with 4 mg triamcinolone (35% with cataract progression and 41% with the need for IOP-lowering medications). During the first year, none of the treated eyes required glaucoma surgery. However, after 24 months, two eyes in the 4 mg IVT group required surgical interventions. SCORE concluded that the therapeutic efficacy was quite similar in two groups although the 4 mg triamcinolone might lead to more adverse effects.

*Dexamethasone Intravitreal Implant (Ozurdex)*

In the GENEVA study, 830 patients from a total of 1,267 patients (66%) with RVO, who had a BRVO for at least six weeks, were randomized to receive dexamethasone implant 0.7 mg, 0.35 mg, or placebo. Eyes with a history of laser photocoagulation were not excluded in this study. Visual improvement of 15 letters or more was achieved faster in both dexamethasone implant groups than the placebo group (P < 0.001). In addition, the percentage of eyes with sustained VA improvement was higher in the dexamethasone implant groups compared to the sham group after the third month (P < 0.001). The therapeutic effect of these implants was not sustained after six months. However, from months 1 to 6, the overall mean increase in VA from baseline was significantly higher in the dexamethasone implant groups compared with the placebo group. In eyes with BRVO-ME with a duration less than 90 days, even greater functional improvement might be achieved with dexamethasone implants comparing to eyes with ME lasting more than three months based on the subgroup post-hoc analysis. The study did not mention CMT in each BRVO subgroups, but overall (i.e., combined CRVO and BRVO) a significant reduction in CMT was seen in both implant groups compared with the sham group in the first three months. However, after six months, anatomical changes did not differ significantly between the groups.^[24]^

Two years later, Guignier et al compared the dexamethasone implant with three monthly IVB injections in the treatment of BRVO-ME. The mean visual gain and CMT reduction was significantly more pronounced at the one-month visit in the intravitreal dexamethasone implant group which was compatible with a faster visual improvement. However, no difference was observed between the two groups at months 3,4, and 6. Despite no difference in mean VA between the study groups at month 6, a significantly higher proportion of eyes treated with bevacizumab gained 15 letters or more compared with the dexamethasone group. More reinjections at month 4 were needed with the dexamethasone implant compared with the IVB treatment.^[55]^

#### Anti-VEGF Agents

*Ranibizumab*. Campochiaro et al^[56]^ published the results of phase III of BRAVO study that compared two ranibizumab doses of 0.3 mg and 0.5 mg with placebo in BRVO-ME. In cases of progressive refractory edema at month 3, rescue grid laser was done. After six months, the BCVA improvement and CMT reduction were higher in both ranibizumab groups compared with the placebo (P < 0.0001).^[57]^ After six months of initial monthly dosing, patients were switched to a PRN injections; 0.5 mg of ranibizumab was injected in the placebo group, while the 0.3 mg and 0.5 mg groups were treated with their original doses. BCVA improvement at six months was maintained in the ranibizumab groups after one year. After starting ranibizumab in the sham group, vision improved; however, this improvement was less than the visual gain in the ranibizumab groups (P < 0.01). The mean CMT change was higher in the 0.3 mg and 0.5 mg groups compared to the sham group (P < 0.05) at six months.

HORIZON study^[31]^ was a two-year extension trial of the BRAVO and CRUISE studies. In the BRVO arm of the study, 304 patients were given evaluation at three-month intervals and were candidates for 0.5 mg ranibizumab if recurrence of ME or a drop in VA was identified. HORIZON study was not completed and led to the variable follow-up periods among the study patients; about 63% of patients (n = 205) from BRAVO trial completed the period of 12 months in the HORIZON study. The mean number of injections in each of the previous BRAVO groups ranged from 2.0 to 2.4 during 12 months of the trial. Visual acuities were significantly increased compared with the BRAVO baseline BCVA in each treatment group. However, considering the baseline values of the HORIZON study, no change or even slight decline in VA was observed in the second year. The study suggested that maximal VA improvement that can be achieved by monthly injections may decline slowly with decreased follow-up visits.

Comparison of 0.5 mg ranibizumab with or without adjunctive macular laser therapy, and laser therapy alone was done by Tadayoni et al.^[58]^ Three monthly intravitreal ranibizumab injections were performed in the ranibizumab groups and subjects were subsequently re-treated PRN according to the designed protocol. At six months, both ranibizumab groups showed significant visual improvement compared to the laser only group (P < 0.0001). There was no difference in the number of injections between the two ranibizumab groups. Anatomically, the mean CMT reduction at six months was significantly more in the ranibizumab groups than the laser group (P < 0.0001).

ITan et al^[59]^ compared intravitreal 0.5 mg ranibizumab with grid laser in BRVO-ME over one year. At the fourth and sixth months, more patients in the grid laser group (68.4% and 50.0%) received additional grid laser compared with the ranibizumab group (6.7% and 8.3%). They concluded that significant and sustained functional and anatomic improvements were provided by the intravitreal ranibizumab in eyes with BRVO-ME.

In the Ranibizumab for Branch Retinal Vein Occlusion Associated Macular Edema Study (RABAMES), patients with BRVO-ME were randomized to treatment with three monthly intravitreal 0.5 mg ranibizumab injections or grid laser or both 60. The mean BCVA improvement after six months was significantly higher in the ranibizumab group compared to the other groups. The combination group required fewer laser retreatments at month 2 compared with the grid laser group (20% versus 70%). They concluded that ranibizumab might be more efficient in visual improvement compared to grid laser photocoagulation. Laser combined with ranibizumab neither augmented VA gain and macular thickness reduction nor did it prevent recurrence of ME. CMT increased gradually after stopping the injections in ranibizumab groups, while visual improvement was sustained, indicating that functional deterioration may occur after the structural disintegrities. Reinjection of ranibizumab after the initial loading dosage, even with a proper response to treatment, may be necessary based on the visual and anatomical changes.

*Bevacizumab*. Multiple studies^[61,62,63]^ evaluated the efficacy of bevacizumab in BRVO-ME although most of them are retrospective or with small sample size. Based on a prospective study by Russo et al,^[63]^ 30 eyes with BRVO were randomized to treatment with monthly IVB or macular grid laser over 12 months. At all time-points of the study, more improvement in BCVA and CMT was observed in the bevacizumab group than the laser group (P < 0.005).

In a prospective randomized interventional study,^[64]^ PRN IVB and subthreshold grid laser were compared as second line therapy for ME in BRVO. This study showed better anatomic and functional results in the IVB group compared to the subthreshold grid laser group.

In a prospective randomized clinical trial by MARVEL group, the efficacy of IVB and ranibizumab (IVR) in BRVO-ME was compared. The number of injections was not significantly different between the treatment groups (3.2 ± 1.5 versus 3.0 ± 1.4, respectively; P = 0.55). There was a significant improvement in VA and CMT in eyes that underwent either bevacizumab or ranibizumab injection without any significant difference between the two drugs.^[65]^

In one study, patients with BRVO-ME were randomly assigned to receive 4 mg IVT monotherapy (n = 17), 1.25 mg IVB monotherapy (n = 14), or a combination of 2 mg IVT and 1.25 mg IVB (n = 21). After one month, all groups showed significant improvement in VA and CMT. At six months, the significant reduction in the CMT was sustained while only the bevacizumab monotherapy group demonstrated significant improvement in the BCVA. Based on these results, at six months, IVB might lead to better functional outcomes compared to the other regimens in this study. Ocular side effects occurred more frequently in the 4 mg triamcinolone group compared to the other groups. A mean IOP increase of 1.4, -0.1, and 0.5 mmHg occurred in three groups, respectively. Also, cataract progression occurred 36%, 8%, and 10% in the three study arms, respectively.^[66]^

In a study by Higashyama,^[67]^ eyes with BRVO-ME were randomly allocated to receive 4 mg IVT or 1.25 mg IVB with 12-month follow-up; additional injections were administered between 3 and 12 months if it was indicated based on the study criteria. After 12 months, the functional improvement from baseline was significantly higher in the IVB group, although no significant difference was seen in CMT reduction between the groups. Therefore, Intravitreal injection of bevacizumab may be a better treatment than that of triamcinolone acetonide for BRVO-ME. Given the three-month delay in resuming the treatment after the initial injection, under-treatment of patients should be considered in the appraisal of the results of this study.

*Aflibercept*. VIBRANT Study^[68]^ (study to Assess the Clinical Efficacy and Safety of VEGF Trap-Eye in Patients with BRVO) was a double-masked randomized trial that evaluated the six-month outcomes of intravitreal aflibercept versus macular laser in BRVO-ME. After six months, monthly aflibercept injection was associated with more functional and structural improvements than macular grid laser in eyes with BRVO-ME.

In the second phase of this study, after the first six months, aflibercept injection was allowed for patients primarily randomized to laser.^[69]^ Aflibercept was injected every eight weeks instead of every four weeks in the primary aflibercept group. It was interesting that even with the eight-week interval regimen, the functional and anatomical improvements gained with monthly injections during the initial six months of the study were maintained. In addition, the laser/aflibercept group had significant VA improvement after initiating aflibercept; however, the improvement was still significantly less than in the eyes that received aflibercept from the beginning of the study (P = 0.02), indicating the importance of earlier initiation of treatment. At week 52, the mean CMT reduction from baseline was -283 and -249 μm in the aflibercept and laser/aflibercept groups, respectively. In conclusion, anatomical outcome was not significantly changed even with deferral of the treatment, but maximum visual improvement which could be achieved with early aflibercept injection might not be attained in the deferral group.

### Meta-analysis

####  Ranibizumab versus Sham Injections

*Participants*. A total of 555 patients with RVO-ME were enrolled in three studies including ROCC,^[28]^ BRAVO,^[56,57]^ and CRUISE.^[29,30]^ The baseline characteristics of participants in these trials were not significantly different concerning age, gender, and systemic factors (e.g., Ischemic heart disease, hypertension, and diabetes mellitus).

**Figure 2:**
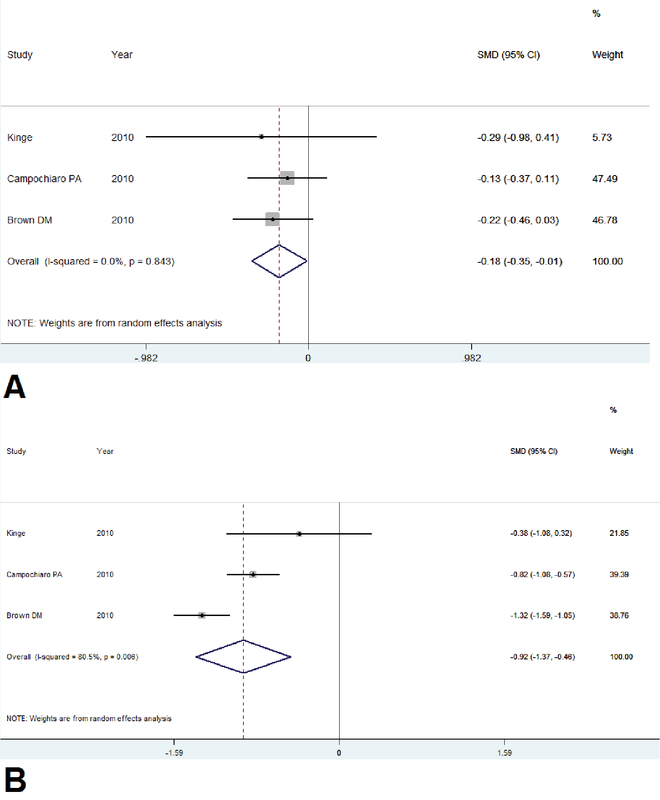
Forest plot displaying pooled summary estimates with ranibizumab treatment versus sham injection at 24 weeks. (a) Regarding VA, there was no significant heterogeneity (I2 = 0%, p = 0.84). Due to small number of included studies, either fixed-effect or random-effect analysis was applied or the overall 95%CI was meaningful (-0.35 to -0.01). (b) Regarding CMT, there was significant heterogeneity (I2 = 80%, p < 0.01), hence, the random-effect model was used and the overall 95%CI was meaningful (-1.37 to -0.46). CI, confidence interval; CMT, central macular thickness; VA, visual acuity

*Mean Change in BCVA and CMT at Six Months*. All three studies reported changes in BCVA and CMT and reported measures of dispersion (SD or 95% CI). At month 6, the pooled mean change in BCVA ranged from -2 to +7.3 letters and +11 to +18.3 letters in the sham and treatment groups, respectively. The highest gain in VA was observed within two months of treatment with anti-VEGFs, with no deterioration thereafter to six months in all studies. After six months, the pooled MD between anti-VEGF and sham was 12.7 letters (95% CI: 11.00 to 13.2). As the statistical heterogeneity was not considerable (I2 = 0%, P = 0.84), data was combined in the meta-analysis because the direction of effect was similar for all trials [Figure 2a]. Results showed superiority of ranibizumab compared to the sham.

The pooled mean CMT improvement at six months ranged from -117 to -167 μm and -304 to -452 μm in the sham and treatment groups, respectively. Meta-analysis of the data showed that patients who underwent ranibizumab injection had more reduction of pooled mean CMT compared with the sham group, (95% CI: -153 to -284 μm). There was significant statistical heterogeneity (I2 = 80%, p < 0.01) [Figure 2b]. Hence, we used the Random-effect model. This represents that, based on anatomical changes, anti-VEGF treatment is associated with clinically significant benefits compared with sham at six months.

#### Bevacizumab versus Intravitreal Triamcinolone

*Participants*. A total of 149 patients, with ME secondary to RVO, were enrolled in three studies (Ramezani et al,^[21]^ Ding et al,^[22]^ and Cekic^[66]^ et al). The baseline characteristics of participants in these trials were also similar.

**Figure 3:**
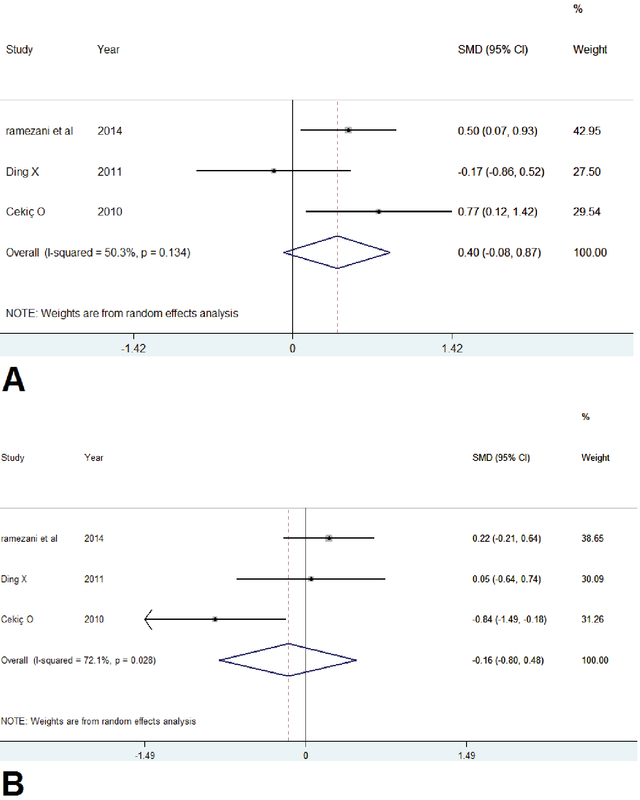
Forest plot displaying pooled summary estimates with bevacizumab treatment versus triamcinolone injection at 24 weeks. (a) Regarding VA, since there was no significant heterogeneity (I2 = 50%, p = 0.13), either fixed-effect or random-effect analysis was applied or the overall 95%CI was not meaningful (-0.08 to 0.87). (b) Regarding CMT, there was significant heterogeneity between included studies (I2 = 72%, p = 0.02) and thus, the random-effect model was used and the overall 95%CI was not meaningful (-0.80 to 0.48). CI, confidence interval; CMT, central macular thickness; VA, visual acuity

*Mean Change in BCVA and CMT at Six Months*. All three studies reported changes in BCVA, CMT, and measures of dispersion (SD or 95% CI) (Ramezani et al, Ding et al, and Cekic et al). The pooled mean change in BCVA letter score at six months ranged from +9 to +48 letters and +23.5 to +32 letters in the triamcinolone and bevacizumab groups, respectively. At six months, the pooled MD between bevacizumab and triamcinolone was 5.3 letters in favor of bevacizumab (95% CI: -16 to 17.5). The statistical heterogeneity was not considerable (I2 = 50.3%, p = 0.13). Since the direction of effect was the same for all of the studies, we combined data in the meta-analysis [Figure 3a]. There was no significant difference in visual improvement between the two therapies.

The pooled mean reduction in CMT at six months ranged from a -75 to -450 μm and -132 to -408 μm in the triamcinolone and bevacizumab groups, respectively. Meta-analysis of the data suggests that the pooled mean CMT reduction was significantly more in the triamcinolone groups compared with the bevacizumab groups during the first month, with no significant differences in macular thickness after six-months follow-up. Patients treated with triamcinolone had a pooled mean reduction in CMT of -68.1 μm more than patients treated with bevacizumab, (95% CI: -58 to -76 μm). There was a significant statistical heterogeneity (I2 = 72.1%, p = 0.02) [Figure 3b]. Therefore, Random-effect model was used. Based on the anatomical changes, no clinically significant superiority of IVB over triamcinolone injections was observed at six months.

##  DISCUSSION

Many pharmaceutical agents have been used for the treatment of RVO-ME; however, three modalities including intravitreal ranibizumab, aflibercept, and dexamethasone implant are FDA approved. The functional and anatomical results of randomized controlled trials of the five most common pharmaceutical agents used in the treatment of RVO-ME are summarized in Tables 1 and 2. Almost all of them could improve the vision and reduce the macular thickness compared with sham injection or macular grid laser photocoagulation at months 6, 12, and 24. In addition, based on seven major RCTs including BRAVO, CRUISE, HORIZON, Epstein et al, COPERNICUS, GALILEO, and VIBRANT, earlier treatment with these agents may lead to better outcomes.^[29,30][31][36,37][43,44,45,46,47,48][56,57][68,69]^ Significant ocular or systemic adverse effects of anti-VEGF agents are rare. Intraocular pressure rise and cataract progression mostly occurred following intravitreal corticosteroid injections.

Based on the SCORE study,^[18]^ the proportion of patients with CRVO gaining 15 letters of ETDRS after 12 months was 6.8%, 26.5%, and 25.6% in the observation, 1 mg IVT, and 4 mg IVT groups, respectively. At four months, the mean reduction in CMT was greater in the 4 mg IVT group. However, no difference was seen regarding CMT improvement among the treatment groups after 12 months. In the triamcinolone-treated patients, authors reported a reduction in ME with a moderate correlation with VA; however, there was a significantly higher percentage of patients requiring IOP-lowering medications and a higher rate of cataract development in the 4 mg IVT group. Hence, the authors suggested 1 mg IVT injection for ME secondary to CRVO. In eyes with BRVO, the comparison was made between intravitreal triamcinolone and laser treatment. No significant difference was found although the 4 mg IVT group again was associated with the highest rate of adverse events.^[18,54]^ Comparing intravitreal triamcinolone and anti-VEGF agents, more functional improvement may occur with anti-VEGF injection although triamcinolone may be accompanied with faster structural improvement.^[21,22,66]^

Dexamethasone implant was compared with the sham treatment in RVO eyes in the GENEVA study.^[23,24][55]^ After three months, both structural and functional improvements were significantly higher in eyes treated with dexamethasone implant compared to sham injection, but this effect was not maintained after six months. The maximum therapeutic effect of intravitreal dexamethasone implant is about 12 weeks after injection. Therefore, treatment repetition is necessary even with dexamethasone implantable agents. The peak response observed at three months is accompanied by the highest incidence of IOP rise and cataract formation. The therapeutic effect (both structural and functional) was greater in eyes with CRVO eyes than in eyes with BRVO.

Patient selection in the GENEVA study was not randomized for retinal ischemia; a large proportion of non-ischemic RVO patients were enrolled. Hence, the study didn't address the use of Ozurdex in ischemic CRVO patients. A post-hoc analysis showed that dexamethasone implantation earlier than three months after the BRVO occurrence was associated with greater VA improvement compared with the eyes with longer duration of ME. According to the COMRADE study,^[26]^ anti-VEGF and dexamethasone implant treatment groups had a similar outcome in the first two months, however, dexamethasone's efficacy decreased from month 3, while ranibizumab maintained its efficacy up to month 6. It showed that dexamethasone re-implantation might be required earlier than six months. The SOLO study also reported early reinjection of dexamethasone implants in most of the cases^[25]^. In the study by Guignier et al, a higher percentage of eyes with BRVO achieved 15 letters or more in the IVB group (30%) compared with the dexamethasone group (11%) after 6 months.^[55]^ Although faster functional and anatomical recovery during the first month was observed in eyes that received dexamethasone implant compared to the IVB-treated eyes, the reinjection rate at four months was also higher with dexamethasone.

In cases undergoing treatment with anti-VEGF injection, various protocols have been suggested. These protocols include loading doses of three to six monthly injections, followed by re-injections on a PRN basis according to the functional and structural changes. It seems that aflibercept injection can be performed at longer intervals without a significant reduction in its efficacy. Although the “treat-and-extend" regimen is frequently applied in anti-VEGF treatment in age-related macular degeneration, this protocol has also been recently used in ME due to RVOs using aflibercept with good outomes.^[51]^ All trials showed that repeated anti-VEGF injection was associated with significant improvement in the functional outcomes at six months compared to the placebo. The functional improvement was also accompanied by favorable structural outcomes.^[18][23,24][29,30][36,37][43,44,45,46,47,48][50][54][56,57][68,69]^ The impact of treatment delay was evaluated in some studies including VIBRANT,^[68,69]^ COPERNICUS,^[43,44,45]^ EPSTEIN,^[36,37]^ and GALILEO.^[64][47,48]^ These RCTs suggested that a shorter interval between the diagnosis and the treatment of RVO-ME was associated with the greatest benefit of anti-VEGFs. The extension of main studies on anti-VEGFs in which the control group received sham injections for six months and then were switched to PRN regimen between months 7 and 12, further confirmed this evidence. Postponing anti-VEGGF therapy for six months could still have a good structural outcome with no significant difference between the treatment groups at month 12. In contrast, while functional outcomes improved with switching to PRN anti-VEGF injections after six months in the sham group, these outcomes remained significantly lower at one year compared with the groups treated with anti-VEGF agents from the outset (COPERNICUS,^[43,44,45]^ CRUISE,^[29,30]^ and Epstein et al^[36,37]^).

In the HORIZON^[31]^ study (the 24-month extension of CRUISE and BRAVO studies), visual outcomes, but not macular thickness, worsened in the second year of treatment especially in the CRVO arm. This may be due to the reduced efficacy of anti-VEGFs during the treatment course. The other reason may be lower treatment frequency from months 12 to 24, especially in patients with CRVO. As we know, the non-perfusion area of retina is usually larger in CRVO than in BRVO, and this causes a higher concentration of VEGF.^[7]^ Therefore, more intravitreal injections may be needed in the second year of treatment of eyes with CRVO-ME than those with BRVO-ME. Regarding iris or retinal neovascularisation or neovascular glaucoma, anti-VEGF therapy led to a significant reduction of neovascular complications compared to the sham treatment at six months (COPERNICUS,^[43,44,45]^ CRUISE,^[29,30]^ Epstein et al,^[36,37]^ GALILEO,^[46][47,48]^ ROCC,^[28]^ and VIBRANT^[68,69]^). In eyes treated with aflibercept, the visual and anatomical improvements that occurred within the first 24 weeks of the study with monthly 2 mg dosing were maintained with bimonthly dosing.

###  Comparison of Studies with Different Treatment Modalities

A direct comparison between CRUISE (ranibizumab) and COPERNICUS/GALILEO (aflibercept), GENEVA (dexamethasone), SCORE (triamcinolone)^[18,24,29,44,47]^ is not appropriate due to several key differences in the study designs, protocols, and population. For instance, eyes with more severity of ischemia (e.g., the presence of an RAPD) and more chronic disease were not enrolled in the BRAVO study but were allowed in SCORE. Hence, it is not surprising to see better results in BRAVO study. Also, longer follow-up periods in the SCORE might have led to under-treatment. Likewise, in the GENEVA study, in the first six months of the study, only a single treatment with dexamethasone implant was allowed, probably resulting in the under-treatment as the implant's peak effect is at two to three months.

In the CRUISE^[29,30]^ trial, patients with sustained ME of more than 12 months or an RAPD were excluded, while prior treatment with anti-VEGF therapy was not defined as an exclusion criteria. In contrast, in COPERNICUS/GALILEO^[43,44,45,46,47,48]^ trials, patients were specifically excluded if they had sustained ME of more than nine-months duration or prior anti-VEGF treatment, and the presence of an RAPD was not mentioned in the exclusion criteria.^[70,71]^

It is also not possible to compare the results between CRUISE and SCORE as CRUISE study might have included healthier eyes compared with SCORE; in CRUISE trial (but not in SCORE), patients were excluded if they had ME for more than 12 months or an RAPD (probably indicating extensive capillary dropout and ischemia); follow-up and re-treatment in CRUISE was monthly, but it was implemented every four months in the SCORE. Therefore, direct comparison of these studies may lead to a misjudgment.^[70]^

Almost all trials reported that the greatest reduction in CMT occurred within a month of the first injection. Also, the CMT improvement was sustained during the treatment period. However, this is in contrary to the observed course of CMT changes in the control groups, who demonstrated a smoother and linear reduction in CMT over the time.

In this study, meta-analysis showed that treatment with ranibizumab was associated with more anatomical and functional improvement compared to the sham after six months, while there was no significant difference in anatomical and visual outcomes between bevacizumab and triamcinolone after six months. Although inclusion criteria for meta-analysis was narrowed to permit including comparable studies with similar arms, it should be noted that some variables (i.e., the time needed to wait before starting the treatment) were not matched and could be a potential source of bias.

##  SUMMARY

This systematic review and the meta-analysis demonstrates that treatment with anti-VEGFs provides significant structural and functional gains compared to the observation in eyes with RVO. There was no significant difference between eyes treated with bevacizumab and intravitreal corticosteroid based on this meta-analysis. However, it seems that repeated anti-VEGF injections, especially for ischemic cases, may be accompanied with better visual outcomes and fewer side effects in the long term.

##  Financial Support and Sponsorship

Nil.

## Conflicts of Interest

There are no conflicts of interest.
